# Morphological and Biochemical Diversity of Shallot Landraces Preserved Along the Croatian Coast

**DOI:** 10.3389/fpls.2018.01749

**Published:** 2018-12-03

**Authors:** Nikola Major, Smiljana Goreta Ban, Branimir Urlić, Dean Ban, Gvozden Dumičić, Josipa Perković

**Affiliations:** ^1^Department of Agriculture and Nutrition, Institute of Agriculture and Tourism, Poreč, Croatia; ^2^The Centre of Excellence for Biodiversity and Molecular Plant Breeding, Zagreb, Croatia; ^3^Department of Applied Sciences, Institute for Adriatic Crops and Karst Reclamation, Split, Croatia; ^4^Department of Plant Sciences, Institute for Adriatic Crops and Karst Reclamation, Split, Croatia

**Keywords:** landrace, mineral composition, morphology, shallot, phenols, PLS

## Abstract

Shallots are a valuable minor *Allium* crop, and are propagated vegetatively and maintained in home gardens across generations along the Croatian coast and island areas. Shallot landraces growing along the Croatian coast fall into three genotypes: *Allium cepa* Aggregatum group (2*n* = 2*x* = 16), *A.* × *proliferum* (Moench) Schard. (2*n* = 2*x* = 16), and *A.* × *cornutum* Clementi ex Vis. (2*n* = 3*x* = 24), among which *A.* × *cornutum* is the most widespread. The aim of this study was to differentiate shallot accessions collected from local farmers using morphological markers. Also, the chemical composition including phenolic content, phenolic profile, total antioxidant capacity, and mineral composition, of shallot accessions was compared with that of the local landraces of common onion, and with market available shallot and common onion cultivars. Based on morphological observations and using multivariate classification, shallot landraces were classified into three distinct groups. Properties, based on which *A. × cornutum* can be differentiated from *A. cepa* Aggregatum and *A. × proliferum*, are stamen morphology, stamen length, leaf and scape vegetative properties, number of bulbs in cluster, cluster mass, and bulb diameter. Flower diameter and flower pedicel length differentiate *A. × cornutum* and *A. × proliferum* from *A. cepa* Aggregatum. Significant variability was observed in the biochemical profiles across tested accessions. Compared with the commercial common onion cultivars, local shallot accessions have higher bulb N, P, and K content. The major phenolic compounds identified in shallots were quercetin-4′-glucoside and quercetin-3,4′-diglucoside. Additionally, several other minor phenolic compounds were also identified. Morphological and biochemical profiles were evaluated using Partial Least Square (PLS) analysis. Specific morphological traits and biochemical markers for possible species identification are proposed.

## Introduction

*Allium* is a taxonomically complicated genus with more than 750 species, and approximately 60 taxonomic groups at subgenera, sectional, and subsectional ranks (Ohri et al., 1998; Fritsch and Friesen, 2002; Block, 2010). Based on inflorescence morphology, *Allium* was once classified as *Liliaceae* and later as *Amaryllidaceae* (Block, 2010). Recently, molecular data have supported further subdivision into small monophyletic families (Fritsch and Friesen, 2002), and placement of *Allium* and its close relatives in the *Alliaceae* family (Takhtajan, 2009).

The origin of the *Allium* spp. is still somewhat a mystery and many botanists doubt the existence of *Allium cepa* as a wild plant (Pike, 1986). Domestication of *Allium* occurred more than 4000 years ago, with spread to Egypt, ancient China, and Persia (Fritsch and Friesen, 2002; Ansari, 2007; Cumo, 2015). *Allium* is currently widely distributed in Europe, Central Asia, North America, and India and shows complex morphological diversity (Stearn, 1992).

*Allium cepa* is one of the oldest cultivated vegetables and is currently the second most widely cultivated vegetable in the world after tomato (FAOSTAT, 2018). Other minor *Allium* species, of less economic importance than onion, are grown sporadically in restricted regions only, and were historically of greater importance (Fritsch and Friesen, 2002). The largest producers of shallots and similar minor *Allium* species are China and Japan, with more than 500,000 tons of shallot bulbs produced per year, followed by New Zealand, Mexico, Iran, Iraq, Cambodia, and Cameroon (FAOSTAT, 2018).

In Croatia, minor *Allium* species are cultivated by local farmers and households along the coastal areas of Istria, Kvarner, Dalmatia, and Dalmatian hinterland. They are generally propagated by bulbs and are closely related to common onions. Recently, Puizina (2013) proposed that shallots in Croatia could be divided into three genotypes based on vegetative and generative morphological characteristics: *A. cepa* Aggregatum (2*n* = 2*x* = 16), *A. × proliferum* (Moench) Schard (2*n* = 2*x* = 16), and *A. × cornutum* Clementi ex Vis. (2*n* = 3*x* = 24), among which *A. × cornutum* is the most widespread in the coastal area. Owing to morphological similarities, it is often difficult to distinguish the species in the field, requiring development of fast and reliable methods for discrimination of landraces to support breeding programs or for commercial exploitation.

Onions are rich in antioxidants, mainly quercetin and its glycosides, and are a major source of dietary flavonoids (Slimestad et al., 2007). In addition, flavonoids are responsible for the yellow or red color of onions (Ferioli and D’Antuono, 2016). Although these health-promoting compounds are ubiquitous in onion bulbs, a detailed chemical profile is required for identification, as the content of specific compounds can vary among *Allium* species or cultivars (Griffiths et al., 2002; Slimestad et al., 2007; Ferioli and D’Antuono, 2016).

Domesticated cultivars, local landraces, ecotypes, or wild edible hybrids are gaining interest, from both economic and nutritional standpoints. The basis for agricultural research, breeding programs, and crop improvement is assessment of plant genetic diversity (Fowler and Hodgkin, 2004; Govindaraj et al., 2015). In the recent years, effort is allocated toward identification and characterization of local landraces in order to preserve the genetic structure from erosion as well as to protect local agronomic production systems by means of agricultural, biological and chemical multidisciplinary approach (Jump et al., 2009; Siracusa et al., 2013; Ferioli and D’Antuono, 2016).

Minor *Allium* crops in Croatia belong to three genetically and morphologically different, vegetatively reproduced relatives of the common onion, *A. cepa* L. (Puizina, 2013). Shallots belonging to *A. cepa* Aggregatum are no longer considered to be a different species, but are classified in the common onion group, as *A. cepa* L. species (Fritsch and Friesen, 2002; Rabinowitch and Kamenetsky, 2002; Brickell et al., 2016).

In this study, shallot accessions collected along the Croatian coast and hinterland were evaluated for their morphological properties. Furthermore, chemical composition of these accessions was compared with that of local landraces of common onions and market-available shallot and common onion cultivars. The diversity observed for the tested traits may be useful for preservation of genetic variability in future breeding programs and to protect local agronomic production systems by means of agricultural, biological, and chemical multidisciplinary approach (Jump et al., 2009; Siracusa et al., 2013; Ferioli and D’Antuono, 2016).

## Materials and Methods

### Material

**FIGURE 1:**
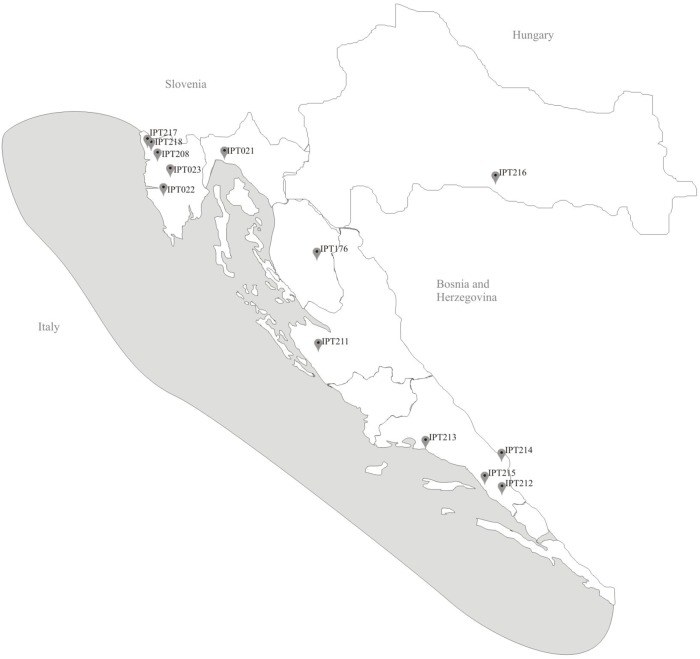
Locations of origin of local shallot accessions.

Shallot landraces were collected from 2014 to 2017 across Croatia (Figure 1) as part of the National Program of Conservation and Sustainable Use of Plant Genetic Resources. Thirteen shallot landraces were collected along Croatian coastal area, from northern and central Istria, Kvarner, Dalmatia, and Dalmatian hinterland areas. The collected landraces were vegetatively propagated by underground bulbs except IPT023 which was propagated by aerial bulbils.

The field trial was established by the end of October 2016 at the Institute of Agriculture and Tourism in Poreč, Croatia (N 45°13′20.30″, E 13°36′6.49″). The shallot clusters consisted of 2–3 bulbs were planted at distance of 20 cm in row and 30 cm between rows. At least 40 clusters of each accession were planted. In addition to shallot landraces, local landraces of common onion were planted as transplants in the same field at the same time at the same planting density (Table 1). Before planting, NPK fertilizer (5:20:30) was incorporated in soil at 500 kg ha^-1^ and at begging of March N was applied (urea source) at a rate of 45 kg ha^-1^. The weeds were removed manually. The plants were grown without irrigation and according to common agricultural practices for onion growing (Lešić et al., 2004). The harvest started at begging of July when at least 50% of pseudo stems bent over for each accession.

**Table 1:** Vegetative and generative morphological qualitative descriptors^1^ of flowering shallot accessions.

Accession	Foliage color (QL1)	Foliage attitude (QL2)	Leaf diameter (QL3)	Cross-section of leaf (QL4)	Degree of leaf waxiness (QL5)	Shape of mature dry bulbs (QL6)	Presence of bulbils (offsets) (QL7)	Number of bulbils (QL8)	Scape^2^ (QL9)	Flower number in umbel (QL10)	Inflorescence^3^ (QL11)	Perianth^4^ (QL12)	Pistil^5^ (QL13)	Stamen morphology^6^ (QL14)	Anther color (QL15)	General fertility (QL16)^7^
***A. × cornutum***																
IPT021	Yellow green	Intermediate	Narrow	Square	Weak	Globe	Present	Few (<30)	1	Many (>30)	3	3	2	3	Yellow	Sterile
IPT022	Yellow green	Intermediate	Narrow	Pentagonal	Medium	Broad elliptic	Present	Few (<30)	1	Many (>30)	3	3	2	3	Yellow	Sterile
IPT211	Yellow green	Intermediate	Narrow	Pentagonal	Weak	Ovate (elongated oval)	Present	Few (<30)	1	Many (>30)	3	3	2	3	Yellow	Sterile
IPT212	Yellow green	Intermediate	Narrow	Pentagonal	Medium	Ovate (elongated oval)	Present	Few (<30)	1	Many (>30)	3	3	2	3	Yellow	Sterile
IPT213	Yellow green	Intermediate	Narrow	Pentagonal	Weak	Ovate (elongated oval)	Present	Few (<30)	1	Many (>30)	3	3	2	3	Yellow	Sterile
IPT214	Yellow green	Intermediate	Narrow	Semi-circular	Weak	Ovate (elongated oval)	Present	Few (<30)	1	Many (>30)	3	3	2	3	Yellow	Sterile
***A. × proliferum***																
IPT023	Green	Erect	Medium Broad	Semi-circular	Weak	Ovate (elongated oval)	Present	Few (<30)	2	Many (>30)	2	2	2	2	Green	Sterile
***A. cepa* Aggregatum**																
IPT208	Yellow green	Intermediate	Medium	Concave	Weak	Broad elliptic	Absent	Absent	1	Many (>30)	1	1	1	1	Green	Fertile
IPT217	Yellow green	Intermediate	Narrow	Circular	Weak	Broad oval	Absent	Absent	1	Many (>30)	1	1	1	2	Green	Fertile
IPT218	Yellow green	Intermediate	Narrow	Circular	Weak	Ovate (elongated oval)	Absent	Absent	1	Many (>30)	1	1	1	2	Green	Fertile


*^*1*^Descriptors in table represent qualitative properties observed on shallot accessions (n = 10) with ability to flower, based on ECP/GR descriptors for vegetatively propagated Allium species and the ones described by Puizina (2013). ^*2*^Scape: conic, hollow, simple (1); conic, hollow, caring bulbils in several levels (2). ^*3*^Inflorescence: round, no bulbils (1); prizmatc, carrying bulbils (2); round, carrying bulbils (3). ^*4*^Perianth: star like, green stripe (1); campanulate, green stripe (2); star like, purple stripe (3). ^*5*^Pistil: lower then stamens (1); taller then stamens (2). ^*6*^Stamen morphology: green, A. cepa type (1); green, simple (2); yellow, A. cepa type (3). ^*7*^QL1 to QL16 are labels of the included qualitative descriptors as seen in Figure 3.*

Commercial cultivars of common onion were purchased at a local market in July 2017, for comparison of biochemical characteristics with those of the accessions in our collection. The cultivars Redwing (red onion), Legend (yellow onion), and Lang Prince de Bretagne (long bulb shallot) were included in the study.

### Morphological Characterization of Local Shallot Landraces

During the vegetative period, the accessions were evaluated according to descriptors for generative organs provided by (Puizina, 2013) and a list of ECP/GR descriptors for vegetatively propagated *Allium* species (IPGRI et al., 2001). Of the 13 coastal shallot accessions collected, only 10 entered reproductive phases, with a flowering period from June 10th to 14th, 2017. These 10 accessions were eligible for morphological differentiation analysis based on flower characteristics. In total, we used 16 qualitative and 10 quantitative plant descriptors for characterization of landraces.

Plants were harvested at maturity and sampled for further analyses after a month of curing in the shade.

### Determination of Macro and Micro Elements

Shallot bulbs were dried in an oven with circulating air at 70°C for 48 h, then ground for nutrient analysis. Powdered material (0.5 g) was obtained from each sample, subjected to dry washing in a muffle furnace at 550°C for 5 h, and used to extract P, K, Ca, Mg, Zn, Mn, and Cu after dissolving in 2 mL HCl. P concentration was determined by the vanadate-molybdate yellow color method (Chapman and Pratt, 1961) using a spectrophotometer at 420 nm. K concentrations were measured using flame photometry (Model 410; Sherwood Scientific Ltd., Cambridge, United Kingdom), while Ca, Mg, Zn, Mn, and Cu were determined by atomic absorption spectrometry (Spectraa 220; Varian Inc., Palo Alto, CA, United States). Total N concentration was measured by the micro-Kjeldahl digestion system (Kjeltec system 1026, Foss Inc., Hilleroed, Denmark).

### Extraction of Soluble Phenolic Compounds

Extraction of phenolic compounds was performed by ultrasound-assisted extraction in 80% methanol. Briefly, 2 g of sample was homogenized with a rotary bearing mill (Model HOMEX 6, Bioreba AG, Reinach, Switzerland) in 9.5 mL of 80% methanol and 0.5 mL NaCl. The mixture was sonicated for 30 min and left to macerate for 4 h at 20°C. The mixture was filtered and centrifuged at 6000 × *g* for 15 min. The resulting supernatant was collected and diluted to a final volume of 10 mL with extraction solvent. The solution was filtered through a 0.45 μm filter prior to analysis.

### Measurement of Total Phenolic Content

Total phenolic content (TPC) was evaluated by the Folin-Ciocalteu assay (Singleton and Rossi, 1965). Sample extracts (0.2 mL) were mixed with 1.4 mL of freshly diluted 0.2 M Folin-Ciocalteu reagent in water. Sodium carbonate (1.4 mL, 6% in distilled water) was added after 1 min and the mixture was vortexed. The reaction mixture was incubated at room temperature and the absorbance of the mixture was read at 750 nm on a UV/Vis spectrophotometer (Model UV-1800, Shimadzu Corporation, Kyoto, Japan). TPC was standardized against gallic acid and expressed as mg of gallic acid equivalents per g sample in fresh weight (FW).

### Quantification of Phenolic Compounds

Chromatographic separations were performed by reversed-phase HPLC. The HPLC instrument consisted of a solvent delivery module (Model ProStar 230, Varian Inc., Palo Alto, CA, United States), a column valve module (Model CVM 500, Varian Inc., Palo Alto, CA, United States), UV/Vis detector (Model ProStar 310, Varian Inc., Palo Alto, CA, United States), and a 5 μm RP C18 column (250 mm × 4.6 mm) (Chromsep Omnispher, Varian Inc., Palo Alto, CA, United States). Gradient elution with solvent A (0.1% formic acid in water) and solvent B (0.1% formic acid in methanol) was achieved using the following program: 90% to 25% A, 0 to 55 min; 25% to 2% A, 55 to 57 min; 2% A, 57 to 69 min. Column temperature was held at 30°C, injection volume was 20 μL, and flow rate was 1.0 mL/min. Individual phenolic compounds were identified and quantified using authentic reference standards of quercetin-3,4′-glucoside, quercetin, isoquercetin, chlorogenic, vanillic, and ferulic acids. Quercetin-4′-glucoside was identified using previously published data and quantified by comparing its relative area with the relative area of the isoquercetin (quercetin-3-glucoside) standard.

### Determination of Total Antioxidant Capacity

Total antioxidant capacity of various *Allium* accessions was evaluated spectrophotometrically (Model UV-1800, Shimadzu Corporation, Kyoto, Japan) by Ferric Reducing Ability of Plasma (FRAP) (Benzie and Strain, 1996) and 2,2-diphenyl-1-picrylhydrazyl (DPPH) radical scavenging assays (Brand-Williams et al., 1995). FRAP values were obtained by analyzing a mixture of 1 mL of sample with 2 mL of freshly prepared FRAP reagent at 593 nm after 4 min of reaction time. Results were expressed as mM of Fe^2+^ equivalents per g sample in FW. DPPH radical scavenging activity was determined by analyzing a mixture of 1 mL of the sample with 2 mL of 0.1 mM DPPH radical at 517 nm after 30 min in darkness. The results were expressed as mM of Trolox equivalents per g sample in FW.

### Statistical Analysis

The morphological description of flowering accessions was conducted on 10 plants per accession as recommended by the IPGRI et al. (2001). Analysis of macro- and micro-elements, phenolic content, phenolic compounds, and antioxidant capacity were performed in triplicate. Data were analyzed by analysis of variance (ANOVA) and Partial Least Square (PLS) analysis using Statistica 13.3 (Tibco, Inc). Significant differences were determined at *p* ≤ 0.05 and homogenous group means were compared by Tukey’s HSD test.

Similarly to Principal Components Regression (PCR), the scope of PLS regression is to form new components that capture most information in the independent variables that is useful for predicting dependent variables, while reducing the dimensionality of the dataset (Garthwaite, 1994). In addition to the information contained in the independent variables, PLS also uses information from dependent variables in the formation of components. As such, PLS is of particular use when there are many independent variables and comparatively little data (Garthwaite, 1994; Helland, 2014). The advantage of PLS regression lies in its exploratory potential. Here the method was applied as an exploratory tool for identification of variables critical in the discrimination between local shallot landraces.

## Results

### Qualitative and Quantitative Morphological Properties

**FIGURE 2:**
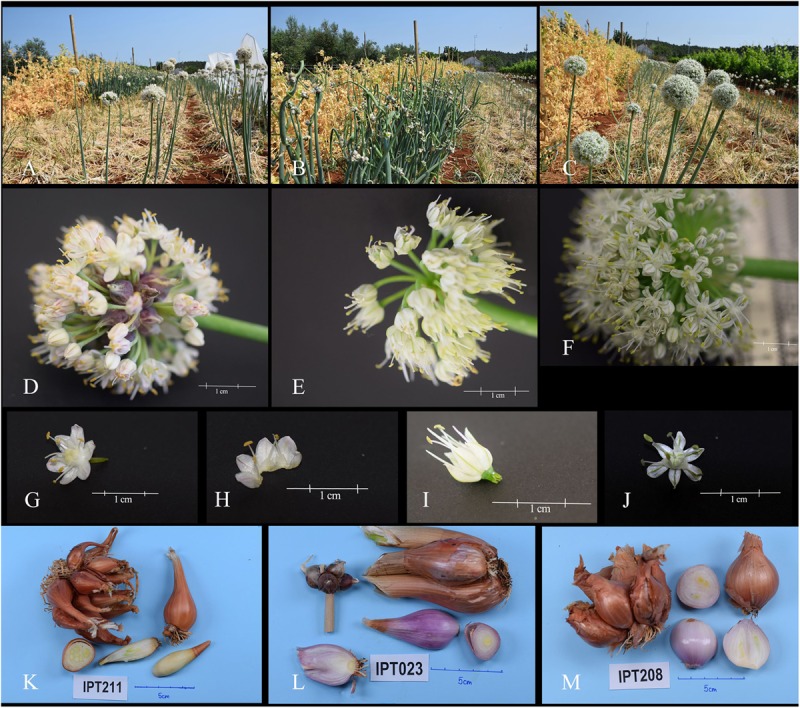
Flowering plant in the field: **(A)** *Allium × cornutum*, **(B)** *Allium × proliferum*, **(C)** *Allium cepa* Aggregatum. Inflorescence: **(D)** *Allium × cornutum*, **(E)** *Allium × proliferum*, **(F)** *Allium cepa* Aggregatum. Flower: **(G,H)** *Allium × cornutum*, **(I)** *Allium × proliferum*, **(J)** *Allium cepa* Aggregatum. Underground bulbs: **(K)** *Allium × cornutum*, **(L)** *Allium × proliferum*, **(M)** *Allium cepa* Aggregatum.

During the growing season, we observed differences in plant habit (Figures 2A–C) and type of inflorescence (Figures 2D–F). Therefore, vegetative and generative plant morphological descriptors were used to describe and group the 10 flowering shallot accessions. Morphological plant descriptors are summarized in Tables 1, 2, and accessions are denoted by their respective species.

**Table 2:** Vegetative and generative morphological quantitative descriptors^1^ of flowering shallot accessions.

Accession	Leaf length (cm) (QN1)^2^	Leaf diameter (cm) (QN2)	Number of bulbs per cluster (QN3)	Cluster mass (g/cluster) (QN4)	Bulb diameter (mm) (QN5)	Scape length (cm) (QN6)	Scape diameter (mm) (QN7)	Inflorescence diameter (mm) (QN8)	Flower pedicle length (mm) (QN9)	Stamen length (mm) (QN10)
***A. × cornutum***										
IPT021	29.60 ± 2.30a^3^	7.56 ± 0.36b	18.40 ± 8.14ab	329.1 ± 48.1bc	34.68 ± 3.93	67.40 ± 1.14bcd	12.79 ± 1.02bcd	45.55 ± 1.66abc	11.58 ± 2.30bc	5.51 ± 0.53c
IPT022	26.50 ± 4.62b	6.70 ± 1.37b	19.00 ± 5.20ab	308.8 ± 88.7bc	33.61 ± 4.69	63.60 ± 5.94bcd	9.68 ± 2.10 cd	41.99 ± 2.07abc	7.91 ± 0.83de	6.94 ± 0.96abc
IPT211	30.84 ± 5.70ab	7.27 ± 0.90b	32.40 ± 12.46ab	404.4 ± 107.2abc	31.37 ± 2.67	64.60 ± 7.27bcd	9.42 ± 1.93cd	30.50 ± 3.00de	7.09 ± 1.31e	5.57 ± 0.92c
IPT212	26.80 ± 3.56b	7.87 ± 2.00b	33.60 ± 21.8a	310.6 ± 114.5bc	34.45 ± 7.95	65.60 ± 7.09bcd	8.10 ± 1.96d	41.43 ± 4.09abcd	8.06 ± 1.04de	6.71 ± 0.92abc
IPT213	28.30 ± 1.52b	6.36 ± 1.21b	30.80 ± 14.50ab	252.0 ± 34.5c	27.85 ± 1.57	53.70 ± 5.65d	8.38 ± 1.36d	35.90 ± 2.38cde	7.05 ± 0.71e	5.72 ± 0.83c
IPT214	31.10 ± 1.67ab	5.68 ± 1.20b	31.00 ± 19.40ab	295.6 ± 119.2bc	29.52 ± 3.50	59.00 ± 8.83cd	8.57 ± 0.85d	38.27 ± 2.70bcde	7.97 ± 0.57de	6.40 ± 0.42bc
***A. × proliferum***										
IPT023	38.10 ± 3.97ab	13.00 ± 1.54a	8.00 ± 1.73b	566.7 ± 147.8a	36.38 ± 2.10	103.20 ± 6.42a	22.88 ± 3.19a	27.91 ± 5.27e	10.57 ± 2.53cd	8.32 ± 1.38a
***A. cepa* Aggregatum**										
IPT208	46.64 ± 7.39a	12.86 ± 3.44a	12.20 ± 1.64ab	483.6 ± 54.5ab	36.26 ± 5.58	66.80 ± 18.19bcd	13.17 ± 3.18bcd	52.12 ± 5.42a	16.51 ± 0.45a	7.84 ± 0.54ab
IPT217	28.80 ± 4.56b	6.34 ± 1.57b	17.40 ± 4.72ab	287.9 ± 69.4c	30.96 ± 4.99	73.00 ± 6.20bc	14.15 ± 1.97bc	38.87 ± 4.82bcde	13.88 ± 1.71ab	6.61 ± 0.77abc
IPT218	32.90 ± 4.16ab	6.27 ± 0.48b	12.20 ± 3.70ab	412.0 ± 66.8abc	30.39 ± 13.52	77.40 ± 6.02b	14.85 ± 4.26b	47.73 ± 12.72ab	15.44 ± 2.21a	6.82 ± 0.65abc
*p*-value	<0.001	<0.001	0.003	<0.001	0,335	<0.001	<0.001	<0.001	<0.001	<0.001


**FIGURE 3:**
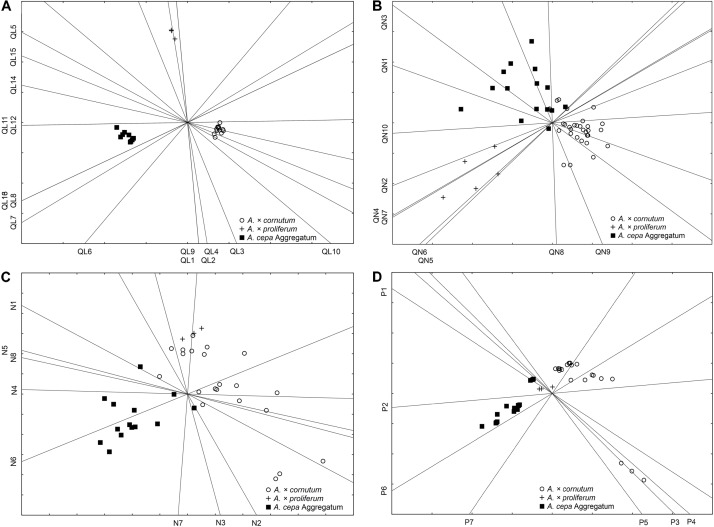
Partial least square (PLS) analysis of local shallot accessions based on **(A)** qualitative morphological descriptors (QL1 to QL16), **(B)** quantitative morphological descriptors (QN1 to QN10), **(C)** nutrient and mineral profiles (N1 to N8), and **(D)** phenolic compounds (P1 to P7).

*^*1*^Descriptors in table represent quantitative morphological properties observed on flowering shallot accessions, based on ECP/GR descriptors for vegetatively propagated Allium species and the ones described by Puizina (2013). ^*2*^QN1 to QN10 are labels of the quantitative descriptors as seen in Figure 3. ^*3*^Data are presented as mean ± SD (n = 10). The different letter within column denotes significant difference by Tukey’s HSD test at p ≤ 0.05.*

The majority of accessions belonged to *A. × cornutum* and were characterized by yellow–green foliage color, intermediate foliage attitude, narrow leaf diameter, and the presence of fewer than 30 bulbils in the inflorescence (Table 1). Scapes of accessions belonging to the *A. × cornutum* group were conic, hollow, simple; round inflorescence with bulbils; perianth purple-green, with pistils taller than stamens; and anthers yellow (Figures 2D,G).

*A. × proliferum* accessions were distinguished from *A. × cornutum* and *A. cepa* Aggregatumg by green foliage color, erect foliage attitude, medium broad leaf diameter (Table 1), prizmic inflorescence, campanulate green striped perianth, and green anther color (Figures 2E,I). Scape morphology in *A. × proliferum* was gigantic in size, carrying bulbils in several levels (Figure 2B), which differed greatly from the other two species.

*Allium cepa* Aggregatum accessions were characterized by circular to concave leaf cross sections; absence of bulbils in inflorescence; star-like, green striped perianth; pistils lower than stamens; green stamens and anthers; and fertile flowers (Figures 2F,J and Table 1).

Although bulbs should be the main organ used to differentiate accessions, their shapes were variable and ranged from elongated oval, broad elliptic, globose, and broad oval to broad elliptic in each of the described accessions (Figures 2K–M and Table 1).

Quantitative morphological characteristics were significantly different among the accessions for all traits studied, except bulb diameter (Table 2). Quantitative differences among *Allium* groups were not as clear as qualitative differences. The gigantic nature of *A. × proliferum* accession (IPT023) was characterized by greater leaf diameter, cluster mass, scape length, and diameter, whereas the bulb number per cluster was generally smaller (Table 2).

Figures 3A,B present PLS analysis of 10 flowering shallot accessions using qualitative and quantitative morphological descriptors presented in Tables 1, 2, respectively. Based on inflorescence (QL11) and perianth (QL12) morphology, all three groups of shallot species could be distinguished from each other (Figures 2D–J, 3A).

*A. × cornutum* shallot accessions could be distinguished from *A. cepa* Aggregatum and *A. × proliferum* based on the degree of leaf waxiness (QL5), flower number in umbel (QL10), stamen morphology (QL14), and anther color (QL15), as shown in Figure 3A.

The Aggregatum group of shallot accessions could be distinguished from the other two species based on the presence (QL7) and number of bulbils (QL8), shape of mature dry bulbs (QL6), and general fertility (QL16), as seen in Figures 2F,J,M, 3A. Furthermore, *A. × proliferum* shallot accessions could be distinguished from *A. cepa* Aggregatum and *A. × cornutum* (Figure 3A) based on extreme vegetative growth (Figure 2B), foliage color (QL1) and attitude (QL2), leaf diameter (QL3) and cross-section shape (QL4), and scape morphology (QL9).

Based on PLS analyses of quantitative descriptors of shallot accessions, it was seen that higher variability separates *A. × proliferum* from the other two species in leaf (QN2) and bulb (QN5) diameter, cluster mass (QN4), and scape length (QN6) and diameter (QN7), confirming gigantism in *A. × proliferum* (Figure 2B). The number of bulbs in clusters (QN3) and leaf length (QN1) are responsible for most of the variability that differentiated *A. × cornutum* from the other two species (Figures 2A,K, 3B). Furthermore, shorter stamen length (QN10) and flower pedicel length (QN9) are the distinguishing factors of *A. × cornutum* (Figure 3B). Inflorescence diameter (QN8) can be used to distinguish *A. cepa* Aggregatum from *A. × cornutum* (Figure 3B), particularly in the case of accession IPT208, as seen in Table 2.

### Nutritional and Mineral Profiles

Mineral profiles of the shallot accessions, commercial onions, and shallot cultivars are shown in Table 3. Based on morphological descriptors, the accessions were assigned to different species. Data showed that *A. × cornutum* was characterized by significantly higher N, Ca, Mg, and Cu content than those in *A. cepa* Aggregatum, but not P and K content (Table 3). The A. × *proliferum* landrace is characterized by significantly lower Mn content than those in *A. × cornutum* and *A. cepa* Aggregatum (Table 3).

**Table 3:** Mineral content of accessions of local shallot landraces and commercial *Allium* cultivars, expressed on FW.

	N (g/kg FW) (N1)^1^	P (g/kg FW) (N2)	K (g/kg FW) (N3)	Ca (g/kg FW) (N4)	Mg (mg/kg FW) (N5)	Zn (mg/kg FW) (N6)	Mn (mg/kg FW) (N7)	Cu (mg/kg FW) (N8)
**Species^2^**								
*A. × cornutum*	5.66 ± 1.06a	0.67 ± 0.01	2.79 ± 0.33	0.73 ± 0.12a	215.1 ± 26.8a	5.82 ± 4.36	1.94 ± 0.31a	1.21 ± 0.35a
*A. × proliferum*	4.48 ± 0.11ab	0.56 ± 0.01	2.83 ± 0.12	0.58 ± 0.02ab	195.3 ± 2.1ab	3.82 ± 0.10	1.11 ± 0.03b	1.18 ± 0.10ab
*A. cepa Aggregatum*	4.85 ± 0.90b	0.72 ± 0.10	2.99 ± 0.26	0.54 ± 0.24b	189.9 ± 14.9b	6.83 ± 4.15	2.10 ± 0.29a	0.95 ± 0.15b
p-value	0.025	0.078	0.143	0.007	0.006	0.489	<0.001	0.030
**Accessions**								
***A. × cornutum***								
IPT021	5.33 ± 0.09cd^3^	0.63 ± 0.01bc	2.70 ± 0.14cde	0.70 ± 0.11abcde	200.0 ± 15.3cd	3.91 ± 0.08	1.41 ± 0.02def	1.17 ± 0.04bc
IPT022	8.12 ± 0.27a	0.91 ± 0.03a	3.41 ± 0.06a	0.82 ± 0.04abc	261.0 ± 8.7a	6.06 ± 0.07	2.39 ± 0.09bc	1.83 ± 0.19a
IPT211	5.57 ± 0.12bc	0.77 ± 0.04ab	2.94 ± 0.09bc	0.84 ± 0.06ab	236.8 ± 10.1ab	4.18 ± 0.32	1.94 ± 0.02bcde	1.51 ± 0.32ab
IPT212	5.51 ± 0.24c	0.67 ± 0.05bc	2.77 ± 0.06cde	0.75 ± 0.03abcd	213.3 ± 6.8bcd	6.28 ± 3.20	2.09 ± 0.02bcd	1.12 ± 0.23bc
IPT213	5.12 ± 0.19cde	0.61 ± 0.01bc	2.76 ± 0.04cde	0.84 ± 0.01ab	220.3 ± 2.8bc	4.19 ± 0.05	2.21 ± 0.08bc	0.91 ± 0.03c
IPT214	5.05 ± 0.07cde	0.52 ± 0.01cd	2.63 ± 0.06de	0.64 ± 0.01bcdef	189.2 ± 2.1de	12.69 ± 13.34	1.84 ± 0.09bcdef	1.02 ± 0.06c
IPT215	4.93 ± 0.06cde	0.60 ± 0.02bc	2.28 ± 0.06f	0.55 ± 0.05defgh	184.7 ± 3.8de	3.39 ± 0.04	1.72 ± 0.06cdef	0.91 ± 0.10c
***A. × proliferum***								
IPT023	4.48 ± 0.11e	0.56 ± 0.01bc	2.83 ± 0.12bcde	0.58 ± 0.02cdefg	195.5 ± 2.1cde	3.83 ± 0.14	1.11 ± 0.03f	1.18 ± 0.10bc
***A. cepa* Aggregatum**								
IPT176	4.86 ± 0.13cde	0.74 ± 0.10abc	2.61 ± 0.04e	0.37 ± 0.05gh	168.0 ± 5.9e	6.58 ± 4.58	1.79 ± 0.09cdef	0.96 ± 0.04c
IPT208	3.72 ± 0.47f	0.76 ± 0.10ab	2.88 ± 0.03bcd	0.94 ± 0.28a	200.3 ± 20.5cd	3.80 ± 0.04	2.07 ± 0.04bcd	0.80 ± 0.03c
IPT216	6.27 ± 0.06b	0.73 ± 0.07abc	3.36 ± 0.07a	0.41 ± 0.05fgh	197.4 ± 7.7cde	13.28 ± 3.34	1.89 ± 0.03bcde	1.10 ± 0.06bc
IPT217	4.73 ± 0.50de	0.76 ± 0.02ab	3.05 ± 0.05b	0.52 ± 0.08defgh	196.6 ± 0.1cde	3.62 ± 0.01	2.17 ± 0.05bcd	0.82 ± 0.19c
IPT218	4.69 ± 0.43de	0.62 ± 0.15bc	3.04 ± 0.03b	0.46 ± 0.01efgh	185.7 ± 3.5de	7.16 ± 5.17	2.57 ± 0.15ab	1.05 ± 0.06c
**Commercial cultivars**								
‘Redwing’	2.23 ± 0.02 g	0.29 ± 0.07e	1.17 ± 0.03 h	0.39 ± 0.10 h	123.8 ± 11.8f	2.26 ± 0.42	2.23 ± 1.15ef	0.85 ± 0.08c
‘Legend’	2.29 ± 0.03 g	0.33 ± 0.02de	0.78 ± 0.06 g	0.47 ± 0.07efgh	113.5 ± 4.3f	2.61 ± 0.03	3.27 ± 0.02a	0.89 ± 0.10c
Long bulbshallot	3.20 ± 0.13f	0.52 ± 0.18cd	2.34 ± 0.23f	0.70 ± 0.07abcde	192.8 ± 23.2cde	9.88 ± 0.92	1.84 ± 0.09bcdef	1.11 ± 0.23bc
*p*-value	<0.001	<0.001	<0.001	<0.001	<0.001	<0.211	<0.001	<0.001


*^*1*^N1–N8 are labels of the included macro- and micro-elements as seen in Figure 3.*

*^*2*^The data represent average of accessions belonging to each species.*

*^*3*^Data are presented as mean ± SD (n = 3). The different letter within column denotes significant difference between accessions of local landraces and commercial cultivars by Tukey’s HSD test at p ≤ 0.05.*

Local shallot accessions had N concentrations from 8.1 ± 0.3 (in IPT022) to 3.7 ± 0.5 g/kg FW (in IPT208), and differed significantly from the values in shallot cultivar Lang Prince (except IPT208) and commercial onion varieties.

The P concentration in commercial shallots did not differ for the majority of shallot accessions, and ranged from 0.5 ± 0.2 g/kg FW (in Lang Prince and IPT214) to more than 0.7 ± 0.1 g/kg FW (in IPT211, IPT216, IPT217, IPT176, and IPT208). Both commercial onion cultivars had much lower P concentrations than that in the local accessions. P concentrations in commercial onions were 2–3 times lower and differed from that in all shallot accessions (local and commercial), where concentrations ranged from 2.34 ± 0.23 g/kg FW (in Lang Prince) to 3.41 ± 0.06 g/kg FW (in IPT022) and 3.36 ± 0.07 g/kg FW (in IPT216). *A. cepa* Aggregatum accession IPT208 had higher Ca concentrations (0.94 ± 0.28 g/kg FW) than that of all accessions belonging to *A. × proliferum* and *A. cepa* Aggregatum. On average, Mg concentrations in fresh bulbs of shallot accessions were twofold higher than in commercial onion varieties. Generally, higher Mg was found in IPT022, and commercial onions had the lowest Mg concentrations when compared with all shallot accessions.

Shallot accessions and commercial samples did not differ significantly in Zn concentrations. The highest Mn in fresh bulbs was found in ‘Legend’ commercial onions, when compared with all other analyzed samples, except IPT218. The highest concentration of Cu was found in IPT022 (1.83 ± 0.19 g/kg) when compared with all other accessions, except IPT211.

Compared with the cultivars of commercial common onions, local shallot accessions had significantly higher N, P, and K levels, while the content of other minerals was not significantly different (Table 3).

The PLS analysis of nutritional and mineral data is shown in Figure 3C. *A. × proliferum* IPT023 and most *A. × cornutum* accessions differed from other groups in P (N2), K (N3), and Mn (N7) content (Figure 3C). Ca (N4) and Zn (N6) represented the largest differences between *A. cepa* Aggregatum and *A. × cornutum*. Mg (N5) and Cu (N8) levels also contributed to differentiation between *A. cepa* Aggregatum and *A. × cornutum*, albeit to a lesser extent owing to comparable levels of these minerals in several shallot accessions from both groups.

### Phenolic Profile and Total Antioxidant Capacity

**FIGURE 4:**
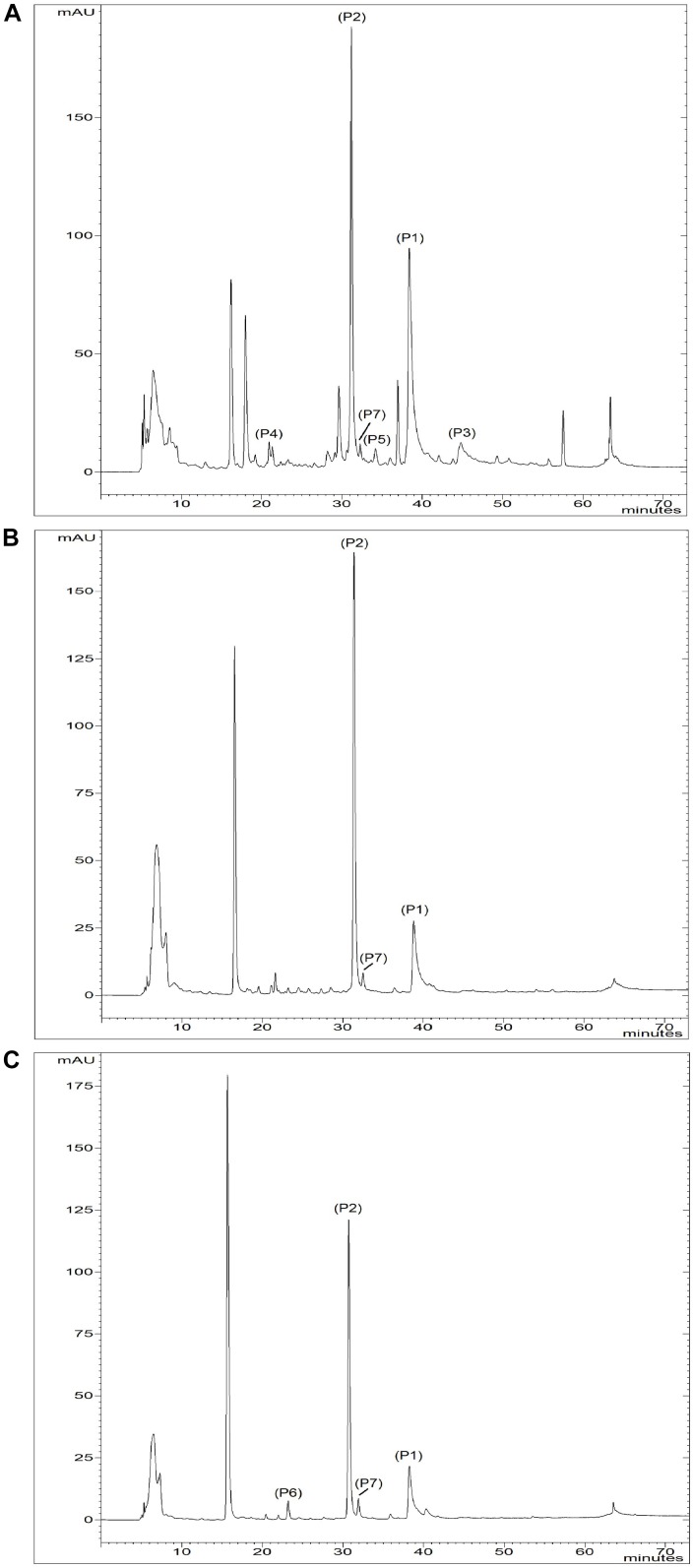
Chromatograms of **(A)** *A. × cornutum* IPT211, **(B)** *A. × proliferum* IPT023, and **(C)** *A. cepa* Aggregatum IPT176 at 280 nm. Peaks: (P1) quercetin-4′-glucoside; (P2) quercetin-3,4′-glucoside; (P3) quercetin; (P4) Chlorogenic acid; (P5) Isoquercetin; (P6) Vanillic acid; (P7) Ferulic acid.

The two most abundant phenolic compounds detected in local shallot accessions were quercetin-4′-glucoside and quercetin-3,4′-diglucoside (Table 4 and Figure 4).

**Table 4:** Phenolic profiles of accessions local shallot landraces, common onion landraces, and commercial *Allium* cultivars expressed in mg/kg FW.

	Quercetin-4′-glucoside (P1)^1^	Quercetin-3,4′-diglucoside (P2)	Quercetin (P3)	Chlorogenic acid (P4)	Isoquercetin (P5)	Vanillic acid (P6)	Ferulic acid (P7)
***Species^2^***							
*A. × cornutum*	337.4 ± 256.5a^3^	168.8 ± 35.8a	26.2 ± 11.7	30.7 ± 5.3	20.8 ± 3.5	n.d.^4^	14.6 ± 0.7
*A. × proliferum*	213.2 ± 52.7ab	124.4 ± 30.8ab	n.d.	n.d.	n.d.	n.d.	14.4 ± 1.5
*A. cepa Aggregatum*	109.3 ± 63.8b	77.7 ± 31.9b	n.d.	n.d.	n.d.	11.1 ± 4.2	19.4 ± 5.5
p-value	0.006	<0.001	–	–	–	–	0.151
**Accessions**							
***A. × cornutum***							
IPT021	133.5 ± 31.0efgh	140.1 ± 28.1de	n.d.	n.d.	n.d.	n.d.	n.d.
IPT022	547.1 ± 104.5b	213.5 ± 39.2a	15.8 ± 3.0b	n.d.	n.d.	n.d.	n.d.
IPT211	845.0 ± 100.9a	191.7 ± 19.3abc	36.6 ± 2.7a	34.5 ± 1.8a	20.8 ± 3.5	n.d.	14.6 ± 0.7c
IPT212	174.8 ± 3.0efg	169.8 ± 6.8abcd	n.d.	n.d.	n.d.	n.d.	n.d.
IPT213	214.5 ± 25.1de	142.6 ± 25.9cde	n.d.	n.d.	n.d.	n.d.	n.d.
IPT214	301.8 ± 13.7cd	194.1 ± 5.8ab	n.d.	25.1 ± 0.3b	n.d.	n.d.	n.d.
IPT215	145.4 ± 22.7efgh	129.4 ± 1.1def	n.d.	n.d.	n.d.	n.d.	n.d.
***A. × proliferum***							
IPT023	213.2 ± 52.7de	124.4 ± 30.8def	n.d.	n.d.	n.d.	n.d.	14.4 ± 1.5c
***A. cepa* Aggregatum**							
IPT176	193.8 ± 22.3def	107.0 ± 15.0efg	n.d.	n.d.	n.d.	10.7 ± 0.9	17.7 ± 1.6b
IPT208	26.2 ± 7.2 h	27.0 ± 5.8hi	n.d.	n.d.	n.d.	n.d.	n.d.
IPT216	67.8 ± 11.4fgh	64.3 ± 11.3gh	n.d.	n.d.	n.d.	14.5 ± 2.5	14.4 ± 0.8c
IPT217	160.5 ± 6.0efg	106.9 ± 3.8efg	n.d.	n.d.	n.d.	8.4 ± 0.4	17.3 ± 0.2b
IPT218	98.2 ± 0.5efgh	83.1 ± 1.5fg	n.d.	n.d.	n.d.	10.0 ± 0.3	28.2 ± 0.3a
***A. cepa***							
IPT003	21.2 ± 5.4 h	10.0 ± 3.4i	n.d.	n.d.	n.d.	n.d.	n.d.
IPT004	29.6 ± 12.8 h	24.3 ± 5.6hi	n.d.	n.d.	n.d.	n.d.	n.d.
**Commercial cultivars**							
‘Redwing’	57.2 ± 4.5gh	30.1 ± 2.2hi	n.d.	n.d.	n.d.	n.d.	n.d.
‘Legend’	222.4 ± 20.1de	121.2 ± 11.5def	n.d.	n.d.	n.d.	n.d.	18.9 ± 0.9b
Long bulbshallot	380.4 ± 58.4c	146.9 ± 13.0bcde	n.d.	n.d.	n.d.	n.d.	n.d.
*p*-value	<0.001	<0.001	<0.001	0.006	–	0.177	<0.001


*^*1*^P1–P7 are labels of phenolic compounds as seen in Figure 3.*

*^*2*^The data represent average of accessions belonging to each species.*

*^*3*^Data are presented as mean ± SD (n = 3). The different letter within column denotes significant difference between accessions of local landraces and commercial cultivars by Tukey’s HSD test at p ≤ 0.05.*

*^*4*^n.d., not determined.*

Quercetin-4′-glucoside and quercetin-3,4′-diglucoside concentration in *A*. *× cornutum* ranged from 845.0 ± 100.9 mg/kg FW (in IPT211) to 133.5 ± 31.0 mg/kg FW (in IPT021) and from 213.5 ± 39.2 (in IPT022) to 129.4 ± 1.1 mg/kg FW (in IPT215), respectively (Table 4). Quercetin-4′-glucoside and quercetin-3,4′-diglucoside concentration in *A. cepa* Aggregatum ranged from 193.8 ± 22.3 mg/kg FW (in IPT176) to 26.2 ± 7.2 mg/kg FW (in IPT 208) and from 107.0 ± 15.0 mg/kg FW (in IPT176) to 27.0 ± 5.8 mg/kg FW (in IPT208), respectively (Table 4). Quercetin-4′-glucoside and quercetin-3,4′-diglucoside concentration in *A. × proliferum* (IPT023) were 213.2 ± 52.7 mg/kg FW and 124.4 ± 30.8 mg/kg FW, respectively, which lie between the quercetin-4′-glucoside levels measured in *A. × cornutum* and *A. cepa* Aggregatum (Table 4).

Quercetin was detected in *A. × cornutum* IPT211 and IPT022 and chlorogenic acid was detected in *A. × cornutum* IPT211 and IPT214 (Table 4). Ferulic acid was detected in *A. × cornutum* IPT211 and *A. cepa* Aggregatum IPT176, IPT216, IPT217, and IPT218 (Table 4).

Vanillic acid (P6) was detected in *A. cepa* Aggregatum IPT176, IPT216, IPT217, and IPT218 (Table 4). In addition, isoquercetin was detected in *A. × cornutum* IPT211, which contained the most abundant and diverse phenolic compound profile of all tested accessions and cultivars (Figure 4 and Table 4).

Local common onion landraces (IPT003 and IPT004) and the ‘Redwing’ commercial common onion had levels of quercetin-4′-glucoside and quercetin-3,4′-diglucoside comparable to those in the accessions of *A. cepa* Aggregatum, except IPT176 (Table 4).

Commercial ‘Legend’ yellow common onions and commercial ‘Lang prince de Bretagne’ long bulb shallots had levels of quercetin-3,4′-diglucoside comparable to those in *A. × proliferum* IPT023, several *A. × cornutum* accessions (IPT021, IPT213, and IPT215), and several *A. cepa* Aggregatum (IPT176 and IPT217) (Table 4).

Average species antioxidant capacities were comparable between *A. × cornutum* accessions and *A. × proliferum*, but were significantly lower in *A. cepa* Aggregatum (Table 5). *A. × cornutum* IPT211 and IPT022 had the highest FRAP and DPPH quenching levels, while the lowest values were measured in local common onion varieties (IPT003 and IPT004) (Table 5).

**Table 5:** Antioxidant capacity and total phenolic content in local shallot landraces, local common onion landraces, and commercial *Allium* cultivars.

	DPPH assay – mM TEQ/FW	FRAP assay – mM Fe2+EQ/g FW	TPC – mg GAEQ/g FW
***Species^1^***			
*A. × cornutum*	1.20 ± 0.34a	2.53 ± 1.46a	1.20 ± 0.37a
*A. × proliferum*	1.23 ± 0.09a	2.09 ± 0.01ab	1.28 ± 0.04a
*A. cepa* Aggregatum	0.73 ± 0.07b	1.34 ± 0.13b	0.80 ± 0.09b
*p*-value	<0.001	0.014	<0.001
**Accession**			
***A. × cornutum***			
IPT021	0.84 ± 0.01fg2	1.50 ± 0.02h	0.99 ± 0.02ef
IPT022	1.62 ± 0.01b	3.31 ± 0.04b	1.47 ± 0.06b
IPT211	1.74 ± 0.01a	5.73 ± 0.08a	1.96 ± 0.01a
IPT212	0.81 ± 0.01fgh	1.54 ± 0.01gh	0.85 ± 0.04ghi
IPT213	1.24 ± 0.02c	1.96 ± 0.01e	1.00 ± 0.05ef
IPT214	1.07 ± 0.03de	1.86 ± 0.03ef	1.14 ± 0.04d
IPT215	1.05 ± 0.01e	1.81 ± 0.02f	1.00 ± 0.04e
***A. × proliferum***			
IPT023	1.23 ± 0.09c	2.09 ± 0.01d	1.28 ± 0.04c
***A. cepa* Aggregatum**			
IPT176	0.77 ± 0.09fgh	1.13 ± 0.06i	0.80 ± 0.02ijk
IPT208	0.64 ± 0.02i	1.23 ± 0.01i	0.81 ± 0.01hij
IPT216	0.81 ± 0.04fgh	1.43 ± 0.07h	0.93 ± 0.03efg
IPT217	0.74 ± 0.02ghi	1.44 ± 0.01h	0.73 ± 0.02jkl
IPT218	0.72 ± 0.02hi	1.42 ± 0.02h	0.71 ± 0.02kl
***A. cepa***			
IPT003	0.42 ± 0.01k	1.15 ± 0.03i	0.36 ± 0.01n
IPT004	0.53 ± 0.01j	1.27 ± 0.03i	0.46 ± 0.02m
**Commercial cultivars**			
‘Redwing’	0.76 ± 0.02gh	1.66 ± 0.02g	0.67 ± 0.01l
‘Legend’	0.89 ± 0.01f	2.09 ± 0.07d	0.91 ± 0.02fgh
Long bulbshallot	1.18 ± 0.05cd	2.40 ± 0.08c	1.11 ± 0.02d
*p*-value	<0.001	<0.001	<0.001


*^*1*^The data represent average of accessions belonging to each species.*

*^*2*^Data are presented as mean ± SD (n = 3). The different letter within column denotes significant difference between accessions of local landraces and commercial cultivars by Tukey’s HSD test at p ≤ 0.05.*

Total phenolic content results reflect phenolic profiles of local accessions (Table 5). In *A. × cornutum*, TPC ranged from 1.96 ± 0.01 mg GAE/g FW (in IPT211) to 0.99 ± 0.02 mg GAE/g FW (in IPT021) (Table 5). In *A. cepa* Aggregatum, TPC levels ranged from 0.93 ± 0.03 mg GAE/g FW (in IPT216) to 0.71 ± 0.02 mg GAE/g FW (in IPT218) (Table 5). In *A. × proliferum*, IPT023 TPC was 1.28 ± 0.04 mg GAE/g FW, which was comparable to TPC levels in *A. × cornutum* (Table 5). *A. cepa* Aggregatum had significantly lower TPC levels than that in *A. × cornutum* and *A. × proliferum* (Table 5). The commercial shallot cultivar and commercial yellow onion ‘Legend’ had TPC levels similar to that in *A*. *× cornutum* accessions; while the commercial red onion cultivar ‘Redwing’ had TPC levels comparable to *A. cepa* Aggregatum accessions (Table 5). Local common onion varieties (IPT003 and IPT004) had the lowest TPC values (Table 5).

Among local shallot accessions, *A. × cornutum* IPT211 had the most abundant and diverse phenolic compound profile (Figure 4 and Table 4). IPT211 also demonstrated high antioxidant capacity, as shown by FRAP and radical scavenging ability, making it the most interesting accession for further studies (Table 5).

Phenolic profile data was processed by PLS analysis to further examine the differences between local shallot accessions (Figure 3D). The property responsible for the most variability was quercetin-4′-glucoside (P1) content, followed by quercetin-3,4′-diglucoside (P2) content (Figure 3D).

Based on quercetin-3,4′-diglucoside (P2) levels, local shallot accessions can be divided into three groups, *A. × cornutum, A. × proliferum*, and *A. cepa* Aggregatum (Figure 3D). Furthermore, *A. cepa* Aggregatum accessions were distinguished from other groups by the presence of vanillic acid (P6), with the exception of IPT208 (Figure 3D and Table 4). *A. × cornutum* had the most variable phenolic profile, as seen with quercetin-4′-glucoside (P1), quercetin (P3), chlorogenic acid (P4), and isoquercetin (P5) content (Figure 3D). These results indicate that local shallot accessions can be discriminated based on their phenolic profiles.

## Discussion

### Qualitative and Quantitative Morphological Properties

In this study, based on morphological observations of reproductive and vegetative plant traits, the accessions belonging to *A. cepa* Aggregatum, (2*n* = 2*x* = 16), *A.* × *proliferum* Moench Schrad. (2*n* = 2*x* = 16), and *A. × cornutum* Clementi ex Vis. (2*n* = 3*x* = 24) were characterized. Among the analyzed accessions characterized using EC/PGR plant morphological descriptors, six belong to *A. × cornutum* (IPT021, IPT022, IPT211, IPT212, IPT213, IPT214, and IPT215), one to *A. × proliferum* (IPT023), and three to *A. cepa* Aggregatum (IPT217, IPT281, and IPT208).

The *A. × cornutum* group is particularly interesting, since it is grown in a relatively narrow coastal region and on islands in Croatia, and has two main common names. In the southern part of the coast (Dalmatia) it is known as ‘Ljutika,’ while in the northern part (Istria) it is known as ‘Škalonja.’ A genetically similar species named ‘Pran,’ can be found in India (Fredotović et al., 2017). Complexity of the triparental origin of allotriploid *A. × cornutum* was previously studied by Friesen and Klaas (1998). However, Puizina et al. (1999) found evidence that two of three parents of triploid viviparous *A. × cornutum* were *A. cepa* and *A. roylei*. Combined molecular phylogenic and cytogenetic studies by Fredotović et al. (2014) provided evidence that the third putative parent of *A. × cornutum* was the wild Asian species *A. pskemense* B. Fedtsch.

Unlike *A. × cornutum, A. × proliferum* is only occasionally found in home gardens. It is a spontaneous hybrid between *A. cepa* and *A. fistulosum* L., and is commonly known as tree onion or Egyptian onion (Puizina and Papeš, 1996; Maass, 1997; Friesen and Klaas, 1998). It is characterized by underdeveloped underground bulbs and very wide diameter of scape, which bares several levels of sprouting bulbils and ends with prizmatic inflorescence.

The shallots of *A. cepa* Aggregatum are more important in the continental region of Croatia (Figure 1, IPT216) (personal observation), and their morphological diversity will be the subject of a future study. These “onion-like” shallots are cultivated around the world, including in Europe, and the same species are known by different common names. In addition, the same common name is sometimes used for different species. Therefore, simple and fast tools for evaluation at the phenological level to provide quick classification on-site for breeders or for curators of genetic banks is potentially very useful.

Partial least square analysis of qualitative and quantitative plant morphological descriptors used in this study confirmed the importance of several qualitative traits for accession characterization (Figures 3A,B). The accessions in this study were clearly separated by inflorescence and perianth morphology. To distinguish *A. × cornutum* from *A. × proliferum*, and *A. cepa* Aggregatum, the degree of leaf waxiness, flower number in umbel, stamen morphology, and anther color were the most important morphological descriptors. *A. × proliferum* was distinguished from the other groups by several qualitative traits, such as foliage color and attitude, leaf diameter and cross-section shape, and scape morphology. In contrast, based on *A. × proliferum* gigantism, quantitative descriptors, such as leaf and bulb diameter, cluster mass, scape length, and diameter might also be employed for discrimination among shallots (Figures 3A,B). *A. cepa* Aggregatum is characterized by lack of bulbils in inflorescence, shape of dry bulb, and fertility, when compared with *A. × cornutum* and *A. × proliferum*. Although the ECP/GR descriptor list is very comprehensive, in the case of a large number of accessions, the shorter list may be utilized for discriminating accessions.

### Nutritional and Mineral Profiles

The main minerals found were N, K, and Ca, while P and Zn were also detected at considerable levels. The mineral content of shallots in our study was similar to values suggested by USDA (2018) for raw shallots, although Ca levels were approximately twofold higher than those reported.

The differences in mineral composition among genotypes and species in our study did not result from differences in cultivation practices or environmental factors, as was suggested for onions and garlic by Ariyama et al. (2006), Petropoulos et al. (2015, 2018), and Vadalà et al. (2016). As the shallot plants were grown in the same field (same soil type and farming practices), the results of our study were because of genotypic differences. The observed differences in mineral content among genotypes may be related to mechanisms controlling nutrient uptake, translocation, or utilization. Our results suggest that commercial common onion cultivars are generally less efficient in nutrient metabolism than local shallot accessions.

The content of phenolic compounds in plant tissues are often negatively affected by high N-nutrition (Treutter, 2010), although experiments with onions showed no significant difference in quercetin-4′-glucoside content between unfertilized onions and onions that received nitrogen fertilizers (Mogren et al., 2007). It is interesting that our accession IPT022 had the highest N concentration in fresh tissue and is among the landraces with higher concentrations of main phenolic compounds. Therefore, it seems that genotype is not related to N-metabolism efficiency or phenolic compound accumulation.

### Phenolic Profile and Total Antioxidant Capacity

The activities of phenolic acids and flavonoids as antioxidants are directly connected to their ability to reduce oxidizing agents, such as free radicals, via functional hydroxyl groups (Wright et al., 2001). Flavonoids are usually present in plants in glycosylated form, resulting in reduced radical scavenging activity, but increased water solubility (Rice-Evans et al., 1997).

As previously reported, the two major phenolic compounds in onion varieties are quercetin-4′-glucoside and quercetin-3,4′-diglucoside (Yang et al., 2004; Bonaccorsi et al., 2005, 2008; Beretta et al., 2017; Fredotović et al., 2017). Soininen et al. (2014) found that long bulb shallot varieties have higher levels of both compounds than round bulb shallot varieties. In our study the content of phenolic compounds was not directly related to bulb shape, since all variety of shapes were found regardless of species. However, when the values for quercetin-4′-glucoside and quercetin-3,4′-diglucoside were averaged for all accessions belonging to same species, we found significantly higher content in *A*. *× cornutum* and *A. × proliferum* than in *A. cepa* Aggregatum.

Several minor compounds were detected in some of the investigated accessions, which helped in their differentiation (Figure 3D). Vanillic and ferulic acids were detected in all investigated *A. cepa* Aggregatum accessions, except in IPT208, which has stamen morphology of *A. cepa* type indicating close genetic similarity. Ferulic acid was also detected in *A. × cornutum* IPT211 and *A. × proliferum* IPT023. Beretta et al. (2017) reported the presence of coumaric and ferulic acids in common onions, and ferulic acid in bunching onions, but not in shallots. Prakash et al. (2007) reported the presence of gallic, ferulic, and protocatechuic acids in four varieties of *A. cepa*. Vanillic, caffeic, ferulic, and chlorogenic acids were detected in addition to the main flavonoids in fresh cut onions, as demonstrated by Chen et al. (2016). In the analyzed samples, chlorogenic acid was detected only in *A. × cornutum* IPT211 and IPT214. The ability to identify and characterize local landraces by phenolic profile has been reported previously in different species, cultivars (Riggi et al., 2013; Lo Bianco et al., 2017), and local landraces of *A. cepa* (Riggi et al., 2013). In our study, the phenolic profile proved to be a powerful tool to discriminate among local shallot accession groups, especially with inclusion of minor phenolic compounds. High levels of the main flavonols, as well as great diversity in minor phenolic compounds suggest *A. × cornutum* IPT211 accession as a prime candidate for further agronomic, genetic, and biochemical studies.

Total phenolic content was determined by the colorimetric Folin–Ciocalteu method, which measures oxidation of phenolic compounds, and the results should correlate well with the estimated antioxidant capacity (Prior et al., 2005). The estimated antioxidant capacity of biological systems should be evaluated using at least two methods to account for interfering compounds (Schlesier et al., 2002; Ozgen et al., 2006). In our study two methods, DPPH free radical quenching and FRAP, were selected. Each rely on electron transfer to determine antioxidant capacity (Prior et al., 2005). In agreement with our study, shallot cultivars commonly have higher levels of flavonoids, TPC, and antioxidant capacity compared with common onion (Yang et al., 2004; Lu et al., 2011; Beretta et al., 2017). Additionally, our results showed that TPC and antioxidant capacity also differ among shallot species found in Croatia, especially *A. × cornutum* and *A. cepa* Aggregatum accessions. It is known that TPC and antioxidant activity in plants change with growing conditions (Heimler et al., 2017), and compound extraction methods (Alothman et al., 2009). A study conducted by Pan et al. (2018) showed that quercetin glucosides in *Allium* species, especially quercetin-4′-glucoside, have great potential as tumor cell growth inhibitors. Higher TPC values in *Allium* methanolic extracts correlated with higher *in vitro* radical scavenging ability and stronger inhibition of tumor cell proliferation (Fredotović et al., 2017). Similarly, in our study, shallot accessions or commercial cultivars with higher TPC exhibited stronger *in vitro* antioxidative effects.

Local landraces are of paramount importance for local agro-economic systems. By providing detailed morphological and chemical characteristics, these landraces can be appropriately preserved and evaluated in addition to the commercial varieties. In this study, shallot accessions important in coastal Croatia were characterized and compared with commercial *Allium* varieties. Local accessions in our study were differentiated according to inflorescence and perianth morphology. The most important morphological descriptors that separated *A. × cornutum* from *A. × proliferum* and *A. cepa* Aggregatum were degree of leaf waxiness, flower number in umbel, stamen morphology, and anther color. *A. × cornutum* and *A. × proliferum* exhibited higher antioxidative capacity and total phenolic content compared with *A. cepa* Aggregatum. *A. × cornutum* is characterized by significantly higher N, Ca, Mg, and Cu content compared with *A. cepa* Aggregatum, while *A. × proliferum* is characterized by significantly lower Mn content. Our results suggest that the investigated landraces possess excellent nutritional qualities, which rival, or even exceed, the quality of commercially developed varieties, especially in terms of the diversity of minor phenolic compounds. The *A. × cornutum* accession IPT211 was found to be of particular interest because of its biochemical wealth and diversity. However, further studies are needed to characterize bioactive constituents in greater depth, which may unravel the benefits and potential new applications of the rediscovered local landraces.

## Author Contributions

DB and SGB designed the experiments. SGB, JP, BU, GD, and NM executed the experiments and analyzed the results. DB, SGB, JP, BU, GD, and NM discussed the results and conclusion of the study. SGB, JP, BU, and NM wrote the manuscript. DB and SGB edited manuscript drafts. All authors approved the manuscript.

## Conflict of Interest Statement

The authors declare that the research was conducted in the absence of any commercial or financial relationships that could be construed as a potential conflict of interest.
